# P044 Mönckeberg’s medial sclerosis as a mimic for giant cell arteritis refractory to treatment

**DOI:** 10.1093/rap/rkag113.074

**Published:** 2026-11-03

**Authors:** Richard Weir, Elizabeth Coulson

**Affiliations:** Department of Rheumatology, Northumbria Healthcare NHS Foundation Trust, Cramlington, United Kingdom; Department of Rheumatology, Northumbria Healthcare NHS Foundation Trust, Cramlington, United Kingdom

## Abstract

**Introduction:**

Mönckeberg’s medial sclerosis (MMS) is a chronic systemic vascular disease characterised by calcium deposition in the medial layer of arterial vessels. The pathology caused by this condition varies depending on sites and vessels affected, and, when affecting the temporal arteries, it can act as a mimic of giant cell arteritis (GCA). We present the case of an 83-year-old Caucasian man whose intermittent headaches posed a diagnostic dilemma and management challenge prior to biopsy confirmation of this rare condition.

**Case description:**

The patient was known to rheumatology after diagnosis of seropositive rheumatoid arthritis aged 81, presenting with intermittent wrist pain, small-joint swelling, and fixed flexion deformities of the right ring and little fingers. Initial investigations showed CRP 65 mg/L, ESR 91 mm/hr, rheumatoid factor 32 kU/L and anti-CCP antibody 32 U/mL. History included IgM kappa monoclonal gammopathy of uncertain significance and normal renal function. Rheumatoid arthritis was initially treated with prednisolone and sulfasalazine, then methotrexate, which was stopped after recurrent shingles.

Four months later, he reported increasingly frequent severe bilateral facial pain, worse on the right, with jaw claudication but no visual symptoms. ESR was 29 mm/hr and CRP 9 mg/L. Examination revealed temporal artery tenderness and a non-pulsatile vessel, raising concern for partially treated giant cell arteritis (GCA), particularly given recent disease-modifying anti-rheumatic drug (DMARD) exposure and long-term steroid use.

Vascular ultrasound showed peri-arterial oedema with positive halo signs in the right frontal and parietal branches and left parietal branch, plus short non-compressible segments in the right frontal and parietal branches. He was assessed as having a high probability of GCA. Biopsy was not performed due to concerns that steroid and DMARD exposure were likely to reduce sensitivity.

Subsequently, the patient experienced a further flare of his symptoms despite high-dose steroid. The treating team agreed strategy for weekly subcutaneous tocilizumab 162mg was started, with a plan to taper prednisolone. After six weeks, he could not reduce prednisolone below 60mg due to persistent scalp tenderness and right-sided jaw claudication.

The case was managed as apparent extremely treatment-resistant GCA. Tocilizumab was changed to intravenous administration, and temporal artery biopsy was performed to explore alternative diagnoses and atypical response. This showed no active inflammation but demonstrated arterial wall thickening consistent with MMS (Figure 1).

**Discussion:**

Following biopsy, tocilizumab infusions were discontinued, and prednisolone was also tapered to stop. Methotrexate was concurrently restarted for control of his rheumatoid arthritis.

Guidance on managing Mönckeberg’s medial sclerosis (MMS) is limited. The clinical team focused on optimising vascular risk factors, as MMS is an independent cardiovascular risk factor and is associated with chronic kidney disease and diabetes. Analgesia was also optimised, with symptoms managed as a daily persistent headache with advice from the neurology team.

This case highlights the diagnostic pitfalls of MMS, which can resemble GCA in clinical history, examination findings, and ultrasound findings. Assessment was further complicated by seropositive rheumatoid arthritis, concurrent DMARD exposure, and long-term steroid use.

Initial concern about biopsy reflected a reasonable high probability of GCA, the patient’s age, and the potential effect of steroid treatment on interpretation. However, temporal artery biopsy ultimately confirmed MMS and clarified the diagnosis.

Temporal artery biopsy was the key in this case to unlock this rare diagnosis and alter treatment strategy accordingly. This case helps underline the importance of tissue biopsy to navigate uncertainty and atypical response even with increasingly sensitive imaging modalities available.

**Key learning points:**

• Mönckeberg’s medial sclerosis (MMS) affecting the temporal artery can closely mimic cranial GCA clinical history, examination, and typical findings on US imaging.

• Ultrasound imaging is increasingly sensitive and more readily available than ever. However, there is still a role for definitive tissue biopsy in GCA in cases of diagnostic uncertainty or atypical cases.

**P044 Figure 1:**
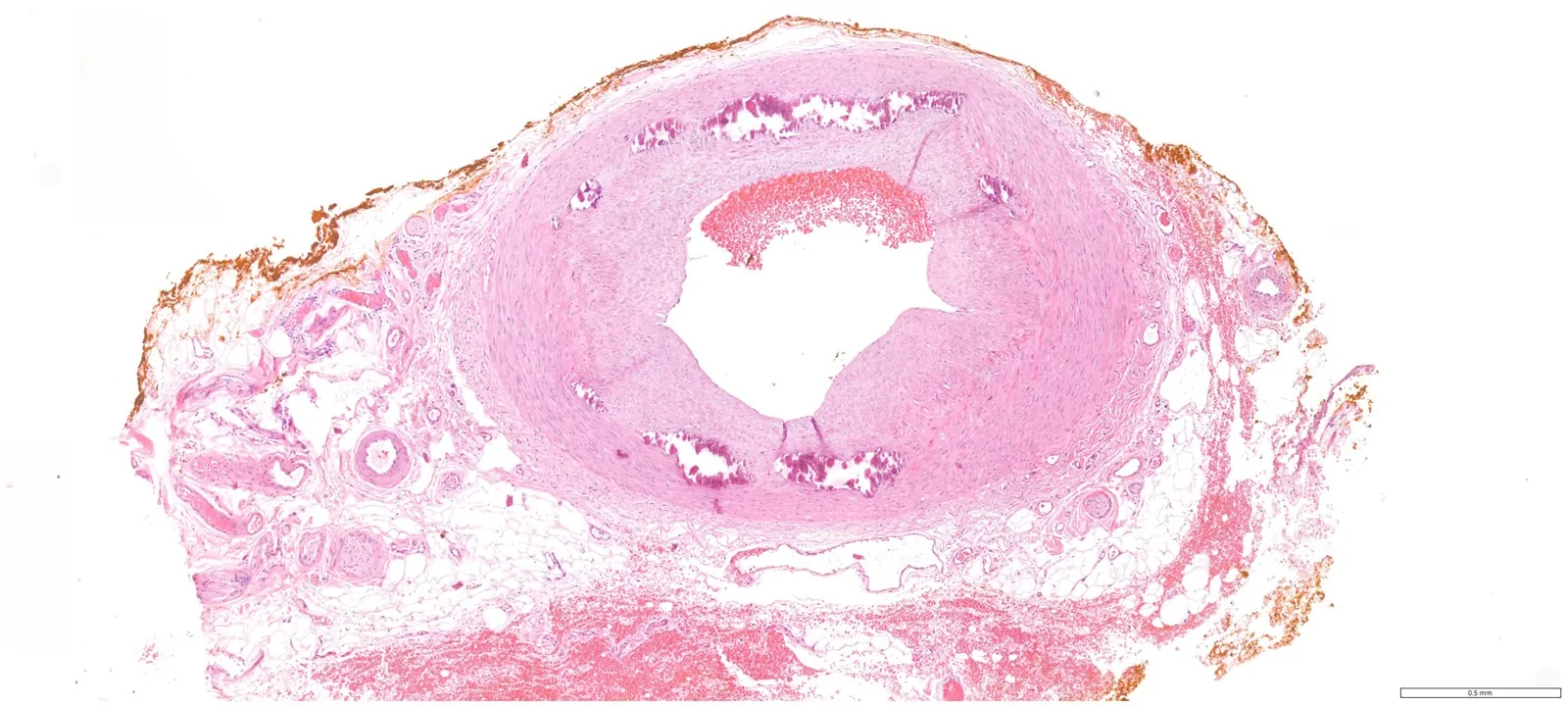
shows a histological slide of the right temporal artery. Microscopic histological examination shows muscular artery with large nodules of calcification within the tunica media, calcification is partially circumferential. No inflammatory activity or giant cells are seen.

